# Improving access to HbA1c in sub-Saharan Africa (IA3) cohort: cohort profile

**DOI:** 10.11604/pamj.2017.27.275.10270

**Published:** 2017-08-13

**Authors:** Naby Balde, Alioune Camara, Joelle Sobngwi-Tambekou, Eric Vounsia Balti, Alain Tchatchoua, Leopold Fezeu, Serge Limen, Sylvie Ngamani, Suzanne Ngapout, Andre Pascal Kengne, Eugene Sobngwi

**Affiliations:** 1 University Hospital Donka, Conakry, Guinea; 2 Catholic University of Central Africa, Yaoundé, Cameroon; 3 Recherche Santé Développement, Yaoundé, Cameroon; 4 Diabetes Research Center, Faculty of Medicine and Pharmacy, Free University of Brussels-VUB, Brussels, Belgium; 5 Universitair Ziekenhuis Brussel- UZ Brussel, Brussels, Belgium; 6 University of Paris 13, Paris, France; 7 South African Medical Research Council and University of Cape Town, Cape Town, South Africa; 8 Faculty of Medicine and Biomedical Sciences, University of Yaoundé 1, Yaoundé, Cameroon

**Keywords:** Diabetes mellitus, HbA1c, diabetes control, complications, mortality

## Abstract

**Introduction:**

Glycated haemoglobin (HbA1c) is the best surrogate of average blood glucose control in diabetic patients, and lowering HbA1c significantly reduces diabetes complications. Moreover, immediate feedback of HbA1c measurement to patients may improve control. However, HbA1c is unavailable in most parts of Africa, a continent with one of the highest burden of diabetes. To translate these evidences, we are conducting a multicentric project in 10 health care facilities in Guinea and Cameroon to evaluate the feasibility and one-year benefit of affordable HbA1c measurement with immediate feedback to patients on diabetes control and related outcomes.

**Participants:**

We consecutively enrolled patients with diabetes mellitus independently of the type of disease. We hypothesised an average 1%-decrease in HbA1c in a 1000-patient study population, with a 20% increase in the number of patients reaching treatment goals within 12 months of intervention and follow-up.

**Findings to date:**

A total of 1, 349 diabetic patients aged 56.2±12.6 years are enrolled (813 in Cameroon and 536 in Guinea) of whom 59.8% are women. The mean duration of diabetes is 7.4±6.3 years and baseline HbA1c is 9.7±2.6% in Guinea and 8.6±2.5% in Cameroon.

**Future plans:**

To investigate whether the introduction of routine HbA1c measurement with immediate feedback to patients and provision of relevant education would improve diabetes control after one year. The impact of the intervention on diabetes associated-complications and mortality warrant further assessment in the long term.

## Introduction

Over the past two decades, consensus has been reached on the pivotal role of HbA1c measurements for the monitoring of diabetes mellitus management strategies [1]. Since the publication in 1993 of the results of the Diabetes Control and Complication Trial (DCCT) showing the tremendous impact of reducing HbA1c in populations of type 1 diabetic patients on the risk of microvascular complications [2], a number of trials have provided similar evidence in other types of diabetes in a variety of settings and circumstances. As a powerful addition to self-monitoring of blood glucose, HbA1c has provided a uniform basis of diabetes control worldwide. It gives both to the patient and his healthcare provider a reliable indication of level of diabetes control over a much longer period compared to other available means of monitoring and at a relatively affordable cost to the patient and to the health systems. However, its measurement tends to rely on heavy techniques such as high performance liquid chromatography that are only available in specialised laboratory settings. These reference methods are time-consuming and technically demanding, with the results of the assay not being available immediately for the purpose of patient education and clinical decision making.

Point-of-care instruments have been developed and validated for the determination of HbA1c in routine clinical care settings. These provide test results within 5-10 minutes for immediate feedback to clinicians and patients, based on less invasive methods such as finger prick capillary blood sample [3]. HbA1c determination with immediate feedback to clinicians and patient enables a perfect alignment of clinical findings with education messages in a one-stop-shop approach. The availability of these new tools has a potential for improving access to HbA1c measurement in resources limited settings where reference methods are likely unavailable. However, just as for any new technology, the feasibility and cost-effectiveness of large scale dissemination of point-of-care HbA1c determination in resource limited countries have not been formally assessed. Improving access to point-of-care based HbA1c testing in resources limited setting has relevance in the sense that randomized controlled trials have demonstrated beneficial effects of immediate feedback of HbA1c test results at the time of patient encounters on short and long terms outcomes of diabetes care [4, 5]. The growing population of people with diabetes worldwide invites innovative approaches to improve the outcome of care and reduce the long term financial burden of caring for chronic complications. Thus, the Improving Access to HbA1c in sub-Saharan Africa (IA3) study was designed on the hypothesis that an intervention that combined clinician education, HbA1c determination with immediate feedback to patients, and targeted patient education would improve the outcome of diabetes care in real life context in sub-Saharan Africa.

**The purpose of the IA3 study**: The objective of the project was to investigate whether the introduction of routine HbA1c measurement with immediate feedback to patients and provision of relevant patient education would improve diabetes control in underserved populations with diabetes clinically diagnosed at least a year prior to the intervention.

## Methods

### Cohort description

IA3 study is based upon baseline assessment of diabetes control and complications, and patient’s knowledge regarding diabetes and its care, a three-monthly assessment of diabetes control with immediate feedback to patient and provision of targeted education, until completion of the planned 12 months follow-up. Thereafter, participants are followed on an annual basis for all-cause mortality. The intervention consists of point-of-care measurement of HbA1c with immediate feedback to patients and provision of targeted education, in addition to usual care. Education is provided in the format of a one-to-one 10-minute session with explanations about what HbA1c is, its clinical and prognostic meaning, and translation of measurement into average blood glucose value. A one-page educational leaflet is also provided at each follow-up visit. HbA1c determination and education are conducted before the routine follow-up consultation.

#### Study settings

**Figure 1:**
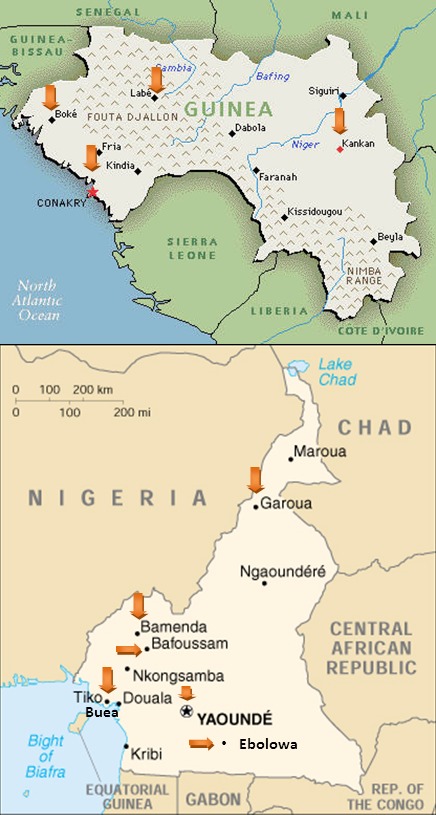
The study sites in Guinea and Cameroon

The study is being conducted in 10 existing diabetes care centres in two sub-Saharan African countries, including 4 regional centres in Guinea, a West African country with 10 million inhabitants, and 6 regional centres in Cameroon, a Central African country with 20 million inhabitants. The health districts covered in Guinea are Conakry, Labe, Boke, Kankan each being situated in a different ecological and cultural areas. In Cameroon, the health districts covered are Yaoundé, Garoua, Buea, Ebolowa, Bamenda, Bafoussam also covering the 4 major ecological zones of Cameroon (Figure 1).

#### Characteristics of the study population

All individuals with confirmed diagnosis of diabetes, irrespective of the type, followed for at least 12 months prior to the study and not intending to migrate from the study site within 1 year were eligible. The 12-month prior follow-up was chosen to reflect usual care to be compared to 12 month intervention in a before and after design. Enrolment of eligible people volunteering to participate was on a consecutive basis during routine consultations in the selected health care facilities after giving their informed consent. In order to detect a difference of 1 unit (1% glycated haemoglobin) between two samples (before and after intervention) with a standard deviation of 1.2%, alpha coefficient of 0.001 and 0.8 power using a two-sided test, a minimum population of 80 subjects is required. The minimum sample size for an alpha of 0.01 is 55 subjects. We therefore decided a sample size of 100 patients per centre to account for possible drop-outs during follow-up, and provide sufficient power for reliable analyses collectively and at the participating centre levels.

#### Data collection

All participants are administered a detailed questionnaire on diabetes history and treatment, coincident risk factors, diet, physical activity, self-care behaviours using instruments previously validated in the target population [6, 7]. In addition, all participants who could read and write in French or English (the official languages of the study countries) filled quality of life, and depression and anxiety questionnaires at baseline. HbA1c is measured at baseline and every 3-month until month 12. Blood samples are collected from all participants at baseline and at month 12, with storage of serum sample at -80^o^C for further assays. Morning urine samples are collected at baseline and at month 12 for the determination of urinary albumin excretion. Urinalysis is performed immediately after collection at baseline and similarly due at month 12. Whole blood on EDTA, and/or saliva samples are collected for all participants for further genomic studies. The primary outcome is the change in HbA1c from baseline, to 3, 6 and 12 months, and secondary outcomes include change in percentage of patients at HbA1c target from baseline to month 12, and change in urinary albumin excretion from baseline to month 12.

## Results

### Preliminary results

A total of 1, 349 (59.8% being women) study participants have been enrolled including 813 in Cameroon and 536 in Guinea. The mean age of the study population is 56.2±12.6 years and the mean duration of diabetes 7.4±6.3 years. Baseline HbA1c is 9.7±2.6% in Guinea and 8.6±2.5% in Cameroon as shown in Table 1.

**Table 1:** Key baseline characteristics overall and by participating centres

	Cameroon						Guinea					
	Yaoundé	Bamenda	Bafoussam	Buea	Garoua	Ebolowa	Conakry	Labé	Boké	Kankan	Total
**N**	410	199	53	56	52	43	335	100	50	51	1, 349
**Percentage women (%)**	56.8	58.3	58.5	67.9	76.9	48.8	61.8	62.00	62.00	54.90	59.8
**Mean Age (yr)**	55.9±12.7	58.6±11.9	51.2±8.5	61.1±9.3	54.1±11.6	56.1±10.6	54.6±13.7	58.6±11.8	57.6±10.9	53.4±14.2	56.2±12.6
**Mean Duration of diabetes (yr)**	7.5±6.9	7.5±5.9	5.3±4.8	6.2±5.4	7.3±6.5	8.0±7.3	7.7±5.8	7.1±5.5	7.5±6.0	7.3±7.5	7.4±6.3
**Mean HbA1c (%)**	8.8±2.6	8.5±2.3	8.2±2.4	8.0±2.2	8.9±2.2	9.0±2.6	9.6±2.6	10.2±2.9	10.0±2.4	9.1±2.6	9.1±2.6

Values are mean and standard deviation or percentage

## Strengths and limitations

The major strengths of this study include the prospective multicentre and bi-country longitudinal nature, the robustness of the design, the translational nature of the research and therefore a direct applicability of the findings to clinical settings. In addition to conducting such an initiative may be for the first time in sub-Saharan Africa, this study may reliably serve as a basis for a larger multi-country cohort study to monitor and compare the outcomes of diabetes care across countries in Africa. The extensiveness of the data collected at baseline, the stored biological samples and the planned long term follow-up are other advantages of the IA3 cohort. The limitations include the lack of control group or non-randomisation (before and after design) and potential for attrition during follow-up [8]. Although difficult to anticipate, this latter limitation has however been accounted for and our current sample size at baseline largely exceeds the adequate size to obtain robust estimates from statistical analysis. Although adequate for the main study purpose the study sample may actually be rather small to investigate major outcomes such as diabetes-associated complications or mortality [9–11]. For such outcomes, much longer duration of follow-up would be needed to accumulate a reasonable number of events.

### Collaboration

Data will be available at the completion of the study upon request to the principal investigators and approval by project board. Biological samples will be available in a centralised repository for further assays, contribution to characterisation of diabetes in sub-Saharan African populations and potential aetiological studies within the limits of the ethical approvals obtained in both participating countries.

## Further details

Additional information is available on the website of IDF-BRIDGES (Bringing Research in Diabetes to Global Environments and Systems) programme (http://www.idf.org/BRIDGES/supported-projects/long-term-1st-round/LT-07-135), Newcastle University, UK (http://www.ncl.ac.uk/ihs/research/project/4008), and upon contact with the corresponding author via e-mail or the website of the Health of population in transition research group in Cameroon (http://www.hopitcam.net).

### Funding

This project is supported by a BRIDGES Grant from the International Diabetes Federation. BRIDGES, an International Diabetes Federation project, is supported by an educational grant from Eli Lilly and Company.

## Competing interests

The authors declare no competing interest.
